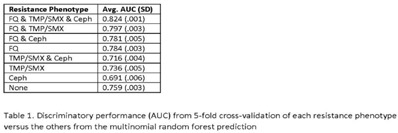# Using machine learning to predict antibiotic resistance to support optimal empiric treatment of urinary tract infections

**DOI:** 10.1017/ash.2022.190

**Published:** 2022-05-16

**Authors:** Ben Brintz, McKenna Nevers, Matthew Goetz, Kelly Echevarria, Karl Madaras-Kelly, Matthew Samore

## Abstract

**Background:** Antibiotic resistance is pervasive in the Veterans’ Affairs (VA) healthcare system, with rates of fluoroquinolone and trimethoprim–sulfamethoxazole (TMP/SMX) resistance approaching 30% in *E. coli* urinary isolates. The efficacy of antimicrobial treatment is critically dependent on the susceptibility of the infecting pathogen; however, prescription decisions are often made empirically in practice. We analyzed susceptibility profiles of enteric gram-negative rods (Enterobacterales) from clinical urine cultures collected from ambulatory patients receiving care in VA clinics and emergency departments. Our goals were (1) to develop a predictive model to support choice of empiric antibiotics pending results of susceptibility testing and (2) to examine the relationship between past antibiotic exposures and susceptibility profiles to enhance understanding of antibiotic selective pressure. **Methods:** We obtained 265,076 positive cultures from 157,422 unique patients from 2015 to 2020. We trained random forest multinomial classifiers to estimate the risk of a positive urine culture isolate being resistant to the multinomial outcome: fluoroquinolone, TMP–SMX, cephalosporin, or any combination of these 3 agents. Data sources evaluated for model generation included demographics, comorbidities, trend and seasonal terms, treatment history for multiple antimicrobial treatments summarized using number of prescriptions in weekly intervals, and sample history summarized by number of resistant and susceptible cultures in weekly intervals. Using 5-fold cross validation, we assess the performance of the clinical prediction using the area under the receiver operating characteristic curve (AUC) for each multinomial outcome. In addition to prediction, we modeled the direct effect of treatment on resistance using multinomial group lasso (MGL). This method allows variable selection in variable groupings, such as all variables related to the fluoroquinolone treatment history, which allowed us to assess the effect of a patient’s complete course of treatment on resistance. **Results:** In cross-validation analysis, our random forest model was best at predicting outcomes with fluoroquinolone resistant phenotypes compared to non–fluoroquinolone-resistant phenotypes (Table 1). From MGL, we found that having a prescription for fluoroquinolone treatment 4–8 weeks prior to a urinalysis was positively associated with fluoroquinolone resistance and negatively associated with fluoroquinolone susceptible phenotypes (Fig. 1). **Conclusions:** Our results show that a patient’s sample and treatment history are highly predictive of a future resistance. Fluoroquinolone treatment is especially associated with increased risk of fluoroquinolone single- and multidrug resistances. A history of either fluoroquinolone or trimethoprim-sulfamethoxazole (TMP-SMX) treatment is a stronger indicator of a future resistant phenotype than cephalosporin or penicillin.

**Funding:** None

**Disclosures:** None

**Figure:**
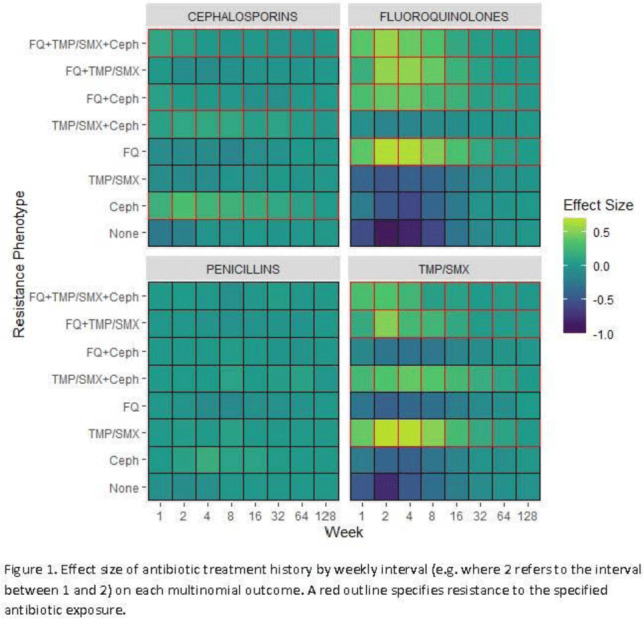

**Table:**